# Arterioarterial Prosthetic Loop as an Alternative Approach for Hemodialysis Access

**DOI:** 10.1097/MD.0000000000001645

**Published:** 2015-10-16

**Authors:** Wenhui Lei, Jiansong Ji, Jian Wang, Lie Jin, Hai Zou

**Affiliations:** From the Department of Nephrology (WL, LJ); Department of Radiology, Lishui Hospital of Zhejiang University, Zhejiang Province (JJ); Department of Vascular and Endovascular Surgery, Lishui hospital of Zhejiang University, China (JW); and Department of Emergency, The Fourth Affiliated Hospital Zhejiang University, Yiwu, People's Republic China (HZ).

## Abstract

In the present study, we performed an arterioarterial prosthetic loop (AAPL) between the femoral artery and deep femoral artery as a new access in patients who did not have adequate vascular conditions for creating an arteriovenous fistula or graft.

Between April 2005 and June 2014, 18 patients received AAPL as a vascular access. During the procedure, a polytetrafluoroethylene graft was anastomosed to the femoral artery and deep femoral artery and looped on the thigh. We assessed the reliability and safety of AAPLs by analyzing complication, primary and secondary patency rates, and postoperative blood flow.

Eighteen patients (median age, 66 years; range, 43–96 years) underwent AAPL access placement under the general or local anesthesia. All patients were followed up for 3 to 38 months (mean, 24 months). Primary and secondary patency rates at 6 months were 94.5% and 88.8%, respectively, and at 3 years were 61% and 72%, respectively. After operation, one patient had infection, and another one had fat necrosis at the surgical incision site. To maintain the AAPL function, 5 surgical procedures in 4 grafts, including revision, thrombectomy, excision, and repair for bleeding were performed. More than 5000 hemodialyses were performed efficiently in our center.

Our study shows that AAPL loop is an unusual but effective and safe procedure that may be a good alternative for the patients who do not allow the conventional hemodialysis access.

## INTRODUCTION

Hemodialysis access has been regarded as a lifeline for the patients with the end-stage renal diseases, and arteriovenous fistula (AVF) is the golden standard access for hemodialysis. According to the Guidelines from Kidney Disease Outcomes Quality Initiative (KDOQI),^1^ all AVF options should be exhausted before resorting to central venous access catheter, an alternative approach for hemodialysis. However, central venous catheter (CVC), compared with AVF, is associated with a greater degree of inflammation in hemodialysis patients, and the catheter itself is associated with a higher mortality.^2^ Moreover, in some patients, because of central vein obstruction such as stenosis and occlusion, CVC cannot be established for hemodialysis, unless radiological and surgical intervention is carried out to remove obstruction.^3^ Therefore, a subset of patients whose vascular access is problematic requires more complicated access procedures.

Because of the aforementioned issues, a synthetic arteriovenous loop graft using the axillary artery and vein in these patients has been reported.^4–6^ When autogenous arteriovenous access is not feasible, the use of prosthetic arteriovenous access for hemodialysis represented a good alternative approach.^7^ When constructed in the right patients, arteriovenous axillary loop grafts permitted satisfactory hemodialysis.^8^ However, in some patients, veins can be exhausted. Thus, a reliable alternative approach should be used for these patients’ hemodialysis.

An artery as a permanent vascular access for hemodialysis is not a new procedure. Butt and Kountz reported^9^ that a femoropopliteal jump graft using a bovine carotid artery as vascular access showed a stable and satisfactory result. Giacchino et al^10^ also reported arterioarterial (AA) jump graft in the upper extremity was a satisfactory hemodialysis access. In 2005, Bunger et al^11^ performed axillary-axillary interarterial chest loop conduit as an alternative for chronic hemodialysis access in 14 patients and achieved an excellent secondary patency rate. The similar technique was used in a 46-year-old woman with a history of complex vascular access in 2013.^12^ Therefore, it is believed that the AA loops may be established when there is no alternative for AV loop.

In 2005, Zanow et al ^13^ used a modified AA loop, arterioarterial prosthetic loop (AAPL), with the proximal axillary or the femoral artery as a vascular access for hemodialysis, which obtained primary and secondary patency rates 73% and 96% at 1 year and 54% and 87% at 3 years, respectively. Since their results were promising, in the present study, we used an AAPL between the femoral artery and deep femoral artery for the patients whose vascular conditions did not allow to have conventional vascular access. Our AAPL is polytetrafluoroethylene (PTFE) graft loop anastomosed with the femoral artery and the deep femoral artery that can be used as vascular access for hemodialysis.

## METHODS

### Patients

From April 2005 to June 2014, AAPL procedures for vascular access were performed in 18 patients with the end-stage renal diseases. All patients who received an AAPL agreed to the procedure by signing a consent form. The committee of human research at Zhejiang University and each study site approved the study.

### Indication

An AAPL for vascular access was recommended only for patients who had no suitable superficial vein (defined as the basilic and cephalic veins) for an AVF and had one of the following indications:

1. An intended AV access or an existing AV access can lead to critical ischemia of the extremity, and this issue is difficult to be solved via other vascular access.
2. Patients could not tolerate the additional cardiac load of a high-flow AV graft due to the cardiac dysfunction, or the AV graft would potentially exacerbate the patients’ congestive heart failure.
3. The large deep veins (defined as basilica, the subclavian, internal jugular and external iliac and femoral veins) were unsuitable for creating an access. A vein was considered unsuitable if an occlusion or high-grade long stenosis (70% in diameter, 3 cm long) in the vein or the venous outflow was detected and could be treated promisingly by any interventions.

Exclusion criteria are ankle brachial indexes <0.8 or superficial femoral artery atherosclerosis.

### Operative Technique

**FIGURE 1:**
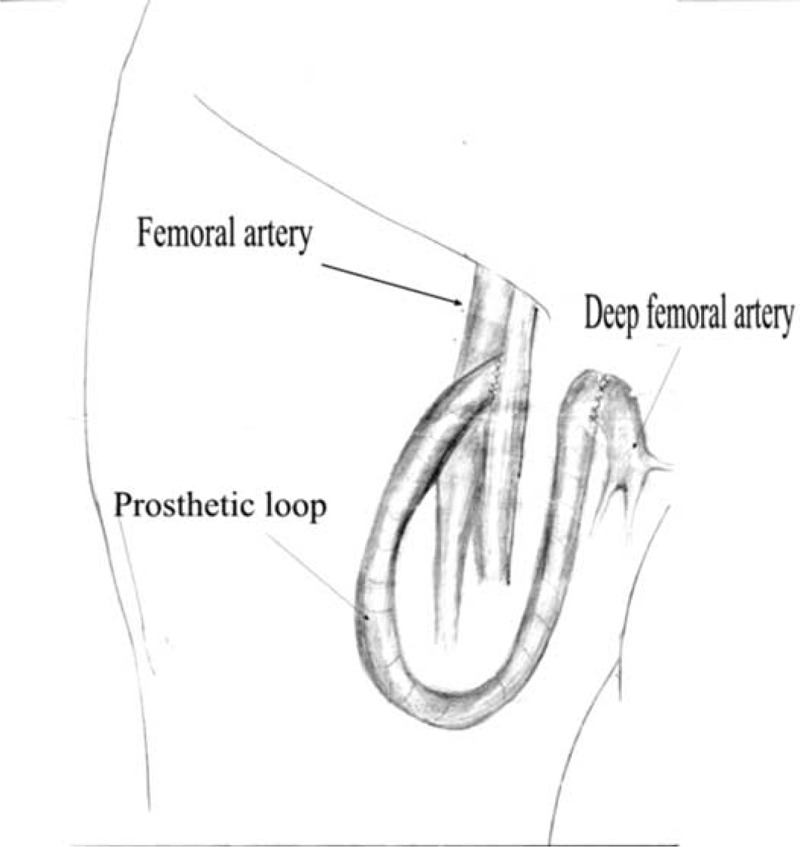
A polytetrafluoroethylene (PTFE) prosthesis loop was anastomosed to the femoral and deep femoral artery.

**FIGURE 2:**
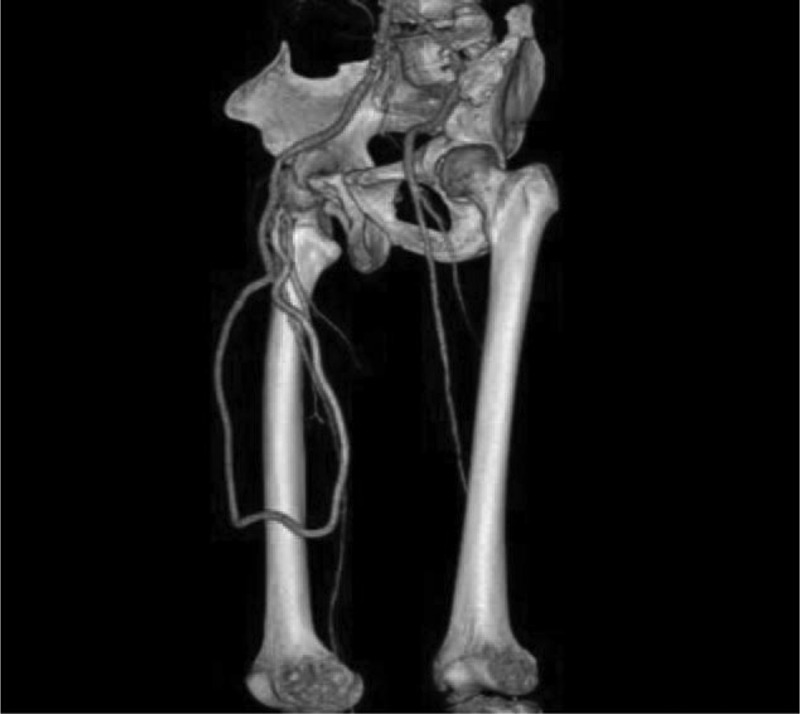
Computed tomography angiography (CTA) of the femoral artery-deep femoral artery arterioarterial prosthetic loop 1 year after placement.

The procedures were performed in the patients under general or local anesthesia, whichever was considered appropriate by the operating team. A diagram of the operative technique for creation of an AAPL access was shown in Figures 1 and 2. The operative procedures included the exposure of the femoral and deep femoral artery, the subcutaneous placement of an expanded PTFE prosthesis with a 6- or 7-mm diameter (adapted to the diameter of the artery) as the loop. After separation of the femoral and deep femoral artery, a PTFE graft was interpositioned after configuration of a subcutaneously tunneled loop on the thigh. A 6–0 polypropylene suture was used in the creation of a side-artery to end-graft anastomoses between the ends of prosthesis and the femoral and deep femoral artery. The length of the implanted graft was between 58 and 60 cm. The mean operation time was 102 minutes, and the blood loss amount was <150 mL.

## MANAGEMENT

### Preoperative Management

All patients were initially assessed through the consultant-led vascular access service at our institution. It was essential to use duplex mapping of arteries and supplementary contrast arteriography to define adequacy or inadequacy of deep arteries. Color duplex ultrasound scanning of arteries was used for all patients, and an arteriography was performed in all patients of suspected arterial inflow or outflow lesions (n = 10). The results of the arteriography showed the femoral and deep femoral artery of all the patients were suitable for AAPL. We chose the better side for creating AAPL and determined a proper PTFE graft loop according to the diameter of the artery. Two grams of cephazolin were given at induction, and low–molecular-weight heparin 4000 U was administered once a day for 3 to 5 days and then was replaced by oral anticoagulation aspirin only (300 mg/day). The first needle puncture of the graft was carried out not before 2 weeks after the procedure.

### Postoperative Surveillance

After discharge, we assessed the graft during dialysis sessions by nursing staff to ensure the patency. We informed the nephrologists about the specifics of this access, and also advised them to adjust the temperature of the reinfused blood, to compress the puncture site for >15 minutes after the removal of the needle, to refrain from any infusion of medications (intra-arterial injection), and to continue the supply of heparin until 30 minutes before finishing hemodialysis. In addition, we provided instructions for the nephrologists when we should change the puncture sites. The needle puncture was performed in only 2 small areas.

All the patients were followed up in our institution every 3 months. We carried out surveillance including graft blood flow (ml/min) and urea reduction ratio (%), clinical examination and duplex ultrasound scanning. If there was any sign of graft dysfunction, Duplex ultrasound was performed as the first-line investigation. A vascular access multidisciplinary meeting was held with surgeons, nephrologists, and radiologists present to discuss any possible interventions to retain patency.

### Statistical Analysis

Statistical analysis was performed using the statistical package SPSS for Windows version13.0 (SPSS Inc, Chicago, IL). Data are presented as mean ± SEM. The Kaplan-Meier method was used to compute survival and graft patency.

## RESULTS

### Demographics

In the past 9 years, 18 PLAAs were performed and were included in our study, which represented about 0.35% of all created vascular accesses for hemodialysis in our institution during this period. This cohort consisted of 10 males and 8 females with a median age of 66 years (range 43–96). Comorbid conditions included coronary artery disease (33%), diabetes mellitus (50%), adipositas (16.6%), hyperlipidemia (55.5%), and documented hypercoagulability (22.22%). All patients had been receiving hemodialysis for 5.3 ± 3.2 years (range, 1–15), with 9 of them for >6 years. They had undergone 14 ± 7 (range, 3 to >21) previous procedures for 6 ± 2 (range, 2–9) with different permanent hemodialysis accesses. The median follow-up time period was 24 months (range, 3–).

The patients were dialyzed through a femoral vein (50%), a temporary CVC placed in the jugular vein (33.4%), or through an insufficient AV graft (16.7%) before the procedure.

AAPL was suggested to be reserved only for selected cases. The patient's clinical characteristics were as follows: 9 patients (50%) had unsuitable large deep veins; 8 patients had endovascular intervention of stenosis of the subclavian or the innominate vein; 1 patient's suitable vein (jugular or femoral vein) was found where a CVC was placed. Two patients had suitable veins, but they all had a severe steal syndrome, with finger necrosis at a low-flow native AVF. The necrosis healed after the construction of the AAPL and the ligation of the AV access.

Nine patients had mixed indications. They had no suitable upper body veins and suitable femoral veins, or coexistent peripheral arterial disease. One patient (16.6%) had a severe congestive heart failure (NYHA class IV, ejection fraction <20%), and was regarded as the indication for an arterial-arterial grafts. Eight patients had central vein obstruction, which was the reason for AAPL.

### Complications

Postoperative leg swelling was the most frequent complication that occurred in 8 patients. The surgery complications 1 month after the procedure were listed as follows: one case had infection at the operative incision site, and the other case had fat necrosis at the operative incision site. There was no thrombogenesis, artery stenosis, vascular infection, heart failure within 30 days’ follow-up. During the early postoperative course, no severe complications were observed. AAPL did not increase the load of heart. Thus, AAPL did not aggravate congestive heart failure in these patients during the follow-up period. The cumulative survival rate for all patients was 88% at 1 year and 72% at 3 years. Five patients died during the follow-up period. However, these patients had a functioning graft being used for dialysis at the time of death.

## INTERVENTION

Seven radiological interventions were performed in 6 grafts, 4 of which were angioplasty of a stenosis, and all of them retained graft patency successfully after intervention. Thrombosis of AAPL occurred in 5 patients (27.7%) at a mean of 16 ± 10 months (range, 6–32) after placement, and all these patients needed thrombectomy combined with the reconstruction of an anastomotic stenosis. Thrombosis of AAPL caused only mild ischemia, but still required immediate thrombectomy to avoid limb ischemia. To maintain the PLAA function, 5 surgical procedures were carried out in 4 grafts, including revision, thrombectomy, excision, and repair for bleeding.

## PATENCY RATES

**FIGURE 3:**
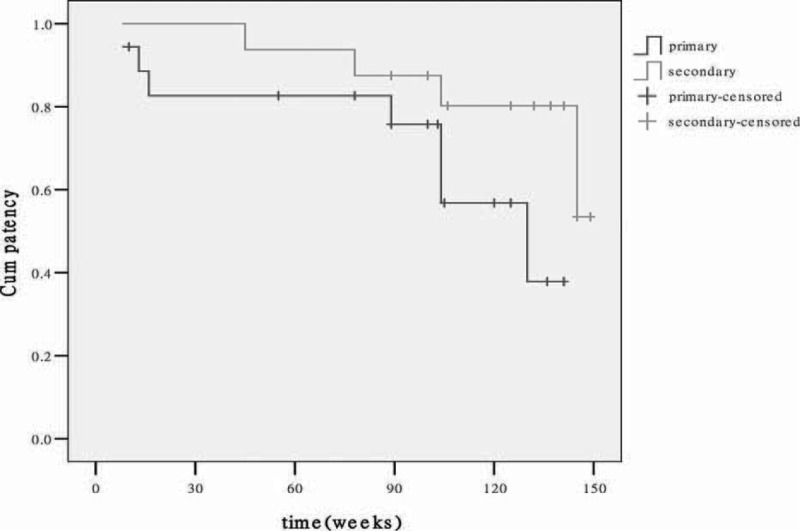
Curves calculated by the Kaplan-Meier method for primary and secondary patency rates.

The primary and secondary patency rates were 94.5% and 88.8% respectively at 6 months; these rates at 3 years were 61% and 72%, respectively. The primary and secondary patency rates were shown in Kaplan-Meier curves (Figure 3).

## DISCUSSION

In the present study, we established AAPL for hemodialysis in those patients who had problems with conventional vascular access. The indication for AAPL application was strictly controlled in our study. We defined 3 main indications for AAPL access: ischemic steal syndrome, exhausted upper extremity access options including central venous occlusion, and cardiac failure. In patients with central venous thrombosis, the thigh AV access could be considered a viable option^14^; however, the failure rate of thigh grafts was high. Right atrial bypass grafting^15^ and axillorenal arteriovenous graft^16^ were described before, but the loop needed anastomosis to the right atrial appendage through a median sternotomy or to the renal vein, and these were thought to be complex access configurations. The arterial-arterial grafts on thigh were also described well previously,^13^ but thrombosis rate of the femoral inguinal arterioarterial prosthetic loop occurred was high. In addition, for the patients without options on upper extremity, prosthetic axillary-axillary arteriovenous straight access was well described in the absence of central vein occlusion.^17^ However, we experienced disappointing results with these procedures in the past. In our study, 8 patients with central vein obstruction could not be recanalized with the smart stents. All of them have failed in constructing an AVF many times. Thus, we selected to develop the arterioarterial thigh access. The symptoms in the patients with ischemic steal syndrome will not vanish until spontaneous AV access occlusion occurred. Indeed, in our study, AAPL was performed in 2 patients with ischemic steal syndrome, and showed an excellent short-term patency rate for the AAPL conduit. The clinical symptoms of these 2 patients were improved after AAPL procedure. For the patients with cardiac failure, the ligation of AVF was necessary. In our study, an exacerbation of congestive heart failure was observed in 1 patient after AAPL construction. This patient died after placement of AAPL for 5 months because of the sudden deterioration of cardiac function.

In the present study, the demographic characteristics of the patients showed a mean age of 66 years and 50% having diabetes. Additionally, most patients had a history of several vein occlusions after temporary CVC placement and multiple failed vascular accesses. Also, arteriosclerosis of the superficial femoral artery is a contraindication to this procedure. Thus, we suggested that duplex ultrasound scanning of artery was mandatory. In addition, since an increased risk of thrombosis after AAPL procedure was assumed, postoperative oral anticoagulation should be routinely applied.

Zanow et al^13^ reported that the basics of AAPL for vascular access in the hemodialysis were different from an AVF. First, a vein was not essential. Second, the distal perfusion was not decreased. Third, the cardiac load was not increased. These were consistent with the observations from our study. However, as mentioned above, one of our patients with heart failure died from sudden deterioration of cardiac function 5 months after AAPL. Thus, some cautions remain to be taken for the patients with existing cardiac failure who are going to undergo AAPL procedure.

Based on our study and pervious reports, we believe that AAPL is another new way to construct the hemodialysis access. Our study demonstrated that the AAPL graft yielded the secondary patency rate at 6 months of 94.5% that was over 70% recommended by the National Kidney Foundation.^1^ Although the secondary patency rate of 72% at 3 years was lower than that reported in previously published studies,^13^ it was still acceptable with regard to the poor vascular conditions and the missing alternative for vascular access.

Although AAPL is a reliable and safe procedure for establishing vascular access for hemodialysis, it should be noted that some complications may accompany it. For example, the thrombosis of a femoral prosthetic loop, if it occurs, requires immediate thrombectomy, or it will lead to distal ischemia. However, in our study, the occlusion of the AAPL was only developed in 1 patient and it was well tolerated. Probably, thrombosis of the deep femoral artery did not lead to a severe distal ischemia because of the good collateral circulation as previously reported.^13^ In addition, graft infection, if it occurs, needs to be treated by removing graft. We did not see any severe infections in our study, only 2 infections that occurred in the surgical incision site. Lastly, AAPL may cause potential problems such as the formation of an aneurysm, embolism, and painful reperfusion.^18^ We observed that 1 patient had embolism, but it did not cause any significant issue. The PLAA may develop a false aneurysm at puncture sites, but it is easy to manage. In fact, if a careful puncture technique is applied, the false aneurysms can be prevented. Zanow et al reported ^13^ that a painful reperfusion was observed in an AAPL with the proximal axillary or the femoral artery as a vascular access for hemodialysis at a dialysis blood flow rate of >400 mL/min, and they believed that the effect was probably caused by the higher pressure on the arterial wall. In our study, the desired sufficient extracorporeal blood flow was 300 mL/min, and the painful reperfusion was not observed.

Despite we have obtained appreciable results in the present study, one limitation of our study was a lack of a control group. Also, given the above-mentioned potential complications, the indications for AAPL must be defined rigidly. The preferable access for hemodialysis remains AVF, and AAPL may be taken as a backup procedure only for patients without any other promising possibility for the creation of a more conventional vascular access.

In conclusion, we demonstrate in the present study that the AAPL is an alternative approach for hemodialysis access and should be considered in those patients who do not have conventional vascular access to hemodialysis. Further long-term follow-up will be required to more accurately assess the outcomes of AAPL and to allow more reasonable comparisons of AAPL with other methods of access.

## References

[1] Clinical practice guidelines for vascular access. *Am J Kidney Dis* 2006; 48 Suppl 1:S176–S247. 16813989 10.1053/j.ajkd.2006.04.029

[2] Banerjee T Kim SJ Astor B Vascular access type, inflammatory markers, and mortality in incident hemodialysis patients: the Choices for Healthy Outcomes in Caring for End-Stage Renal Disease (CHOICE) Study. *Am J Kidney Dis* 2014; 64:954–961. 25266479 10.1053/j.ajkd.2014.07.010 PMC4265216

[3] Dammers R de Haan MW Planken NR Central vein obstruction in hemodialysis patients: results of radiological and surgical intervention. *Eur J Vasc Endovasc Surg* 2003; 26:317–321. 14509897 10.1053/ejvs.2002.1943

[4] Mohamed IH Bagul A Doughman T Axillary-axillary loop graft for hemodialysis access. *J Vasc Access* 2011; 12:262–263. 21218383 10.5301/JVA.2011.6215

[5] Elwakeel H Khafagy T Regal S Prosthetic axillary-axillary arm loop arteriovenous graft for hemodialysis. *Int Angiol* 2013; 32:589–592. 24212292

[6] Kendall TW Jr Cull DL Carsten CG 3rd The role of the prosthetic axilloaxillary loop access as a tertiary arteriovenous access procedure. *J Vasc Surg* 2008; 48:389–393. 18515038 10.1016/j.jvs.2008.03.030

[7] Shemesh D Olsha O Berelowitz D Integrated approach to construction and maintenance of prosthetic arteriovenous access for hemodialysis. *Vascular* 2004; 12:243–255. 15704319 10.1258/rsmvasc.12.4.243

[8] Jean-Baptiste E Hassen-Khodja R Haudebourg P Axillary loop grafts for hemodialysis access: midterm results from a single-center study. *J Vasc Surg* 2008; 47:138–143. 18178466 10.1016/j.jvs.2007.09.059

[9] Butt KM Kountz SL A new vascular access for hemodialysis: the arterial jump graft. *Surgery* 1976; 79:476–479. 1257909

[10] Giacchino JL Geis WP Buckingham JM Vascular access: long-term results, new techniques. *Arch Surg* 1979; 114:403–409. 435054 10.1001/archsurg.1979.01370280057008

[11] Bunger CM Kroger J Kock L Axillary-axillary interarterial chest loop conduit as an alternative for chronic hemodialysis access. *J Vasc Surg* 2005; 42:290–295. 16102629 10.1016/j.jvs.2005.04.022

[12] Stephenson MA Norris JM Mistry H Axillary-axillary interarterial chest loop graft for successful early hemodialysis access. *J Vasc Access* 2013; 14:291–294. 23172167 10.5301/jva.5000121

[13] Zanow J Kruger U Petzold M Arterioarterial prosthetic loop: a new approach for hemodialysis access. *J Vasc Surg* 2005; 41:1007–1012. 15944601 10.1016/j.jvs.2005.02.043

[14] Miller CD Robbin ML Barker J Comparison of arteriovenous grafts in the thigh and upper extremities in hemodialysis patients. *J Am Soc Nephrol* 2003; 14:2942–2947. 14569105 10.1097/01.asn.0000090746.88608.94

[15] El-Sabrout RA Duncan JM Right atrial bypass grafting for central venous obstruction associated with dialysis access: another treatment option. *J vasc Surg* 1999; 29:472–478. 10069911 10.1016/s0741-5214(99)70275-2

[16] Karp SJ Hawxby A Burdick JF Axillorenal arteriovenous graft: a new approach for dialysis access. *J Vasc Surg* 2004; 40:379–380. 15297838 10.1016/j.jvs.2004.03.026

[17] Morsy MA Khan A Chemla ES Prosthetic axillary-axillary arteriovenous straight access (necklace graft) for difficult hemodialysis patients: a prospective single-center experience. *J Vasc Surg* 2008; 48:1251–1254.1254.e1251. 18771891 10.1016/j.jvs.2008.06.064

[18] Rooijens PP Burgmans JP Yo TI Autogenous radial-cephalic or prosthetic brachial-antecubital forearm loop AVF in patients with compromised vessels? *J Vasc Surg* 2005; 42:481–486. 16171591 10.1016/j.jvs.2005.05.025

